# Scalable approaches for functional analyses of whole-genome sequencing non-coding variants

**DOI:** 10.1093/hmg/ddac191

**Published:** 2022-08-09

**Authors:** Pavel P Kuksa, Emily Greenfest-Allen, Jeffrey Cifello, Matei Ionita, Hui Wang, Heather Nicaretta, Po-Liang Cheng, Wan-Ping Lee, Li-San Wang, Yuk Yee Leung

**Affiliations:** Penn Neurodegeneration Genomics Center, Department of Pathology and Laboratory Medicine, Perelman School of Medicine, University of Pennsylvania, Philadelphia, PA 19104, USA; Department of Pathology and Laboratory Medicine, Perelman School of Medicine, University of Pennsylvania, Philadelphia, PA 19104, USA; Penn Neurodegeneration Genomics Center, Department of Pathology and Laboratory Medicine, Perelman School of Medicine, University of Pennsylvania, Philadelphia, PA 19104, USA; Department of Genetics, Perelman School of Medicine, University of Pennsylvania, Philadelphia, PA 19104, USA; Penn Neurodegeneration Genomics Center, Department of Pathology and Laboratory Medicine, Perelman School of Medicine, University of Pennsylvania, Philadelphia, PA 19104, USA; Department of Pathology and Laboratory Medicine, Perelman School of Medicine, University of Pennsylvania, Philadelphia, PA 19104, USA; Penn Neurodegeneration Genomics Center, Department of Pathology and Laboratory Medicine, Perelman School of Medicine, University of Pennsylvania, Philadelphia, PA 19104, USA; Department of Pathology and Laboratory Medicine, Perelman School of Medicine, University of Pennsylvania, Philadelphia, PA 19104, USA; Penn Neurodegeneration Genomics Center, Department of Pathology and Laboratory Medicine, Perelman School of Medicine, University of Pennsylvania, Philadelphia, PA 19104, USA; Department of Pathology and Laboratory Medicine, Perelman School of Medicine, University of Pennsylvania, Philadelphia, PA 19104, USA; Penn Neurodegeneration Genomics Center, Department of Pathology and Laboratory Medicine, Perelman School of Medicine, University of Pennsylvania, Philadelphia, PA 19104, USA; Department of Pathology and Laboratory Medicine, Perelman School of Medicine, University of Pennsylvania, Philadelphia, PA 19104, USA; Penn Neurodegeneration Genomics Center, Department of Pathology and Laboratory Medicine, Perelman School of Medicine, University of Pennsylvania, Philadelphia, PA 19104, USA; Department of Pathology and Laboratory Medicine, Perelman School of Medicine, University of Pennsylvania, Philadelphia, PA 19104, USA; Penn Neurodegeneration Genomics Center, Department of Pathology and Laboratory Medicine, Perelman School of Medicine, University of Pennsylvania, Philadelphia, PA 19104, USA; Department of Pathology and Laboratory Medicine, Perelman School of Medicine, University of Pennsylvania, Philadelphia, PA 19104, USA; Penn Neurodegeneration Genomics Center, Department of Pathology and Laboratory Medicine, Perelman School of Medicine, University of Pennsylvania, Philadelphia, PA 19104, USA; Department of Pathology and Laboratory Medicine, Perelman School of Medicine, University of Pennsylvania, Philadelphia, PA 19104, USA; Penn Neurodegeneration Genomics Center, Department of Pathology and Laboratory Medicine, Perelman School of Medicine, University of Pennsylvania, Philadelphia, PA 19104, USA; Department of Pathology and Laboratory Medicine, Perelman School of Medicine, University of Pennsylvania, Philadelphia, PA 19104, USA

## Abstract

Non-coding genetic variants outside of protein-coding genome regions play an important role in genetic and epigenetic regulation. It has become increasingly important to understand their roles, as non-coding variants often make up the majority of top findings of genome-wide association studies (GWAS). In addition, the growing popularity of disease-specific whole-genome sequencing (WGS) efforts expands the library of and offers unique opportunities for investigating both common and rare non-coding variants, which are typically not detected in more limited GWAS approaches. However, the sheer size and breadth of WGS data introduce additional challenges to predicting functional impacts in terms of data analysis and interpretation. This review focuses on the recent approaches developed for efficient, at-scale annotation and prioritization of non-coding variants uncovered in WGS analyses. In particular, we review the latest scalable annotation tools, databases and functional genomic resources for interpreting the variant findings from WGS based on both experimental data and *in silico* predictive annotations. We also review machine learning-based predictive models for variant scoring and prioritization. We conclude with a discussion of future research directions which will enhance the data and tools necessary for the effective functional analyses of variants identified by WGS to improve our understanding of disease etiology.

## Graphical Abstract

**Graphical Abstract:**
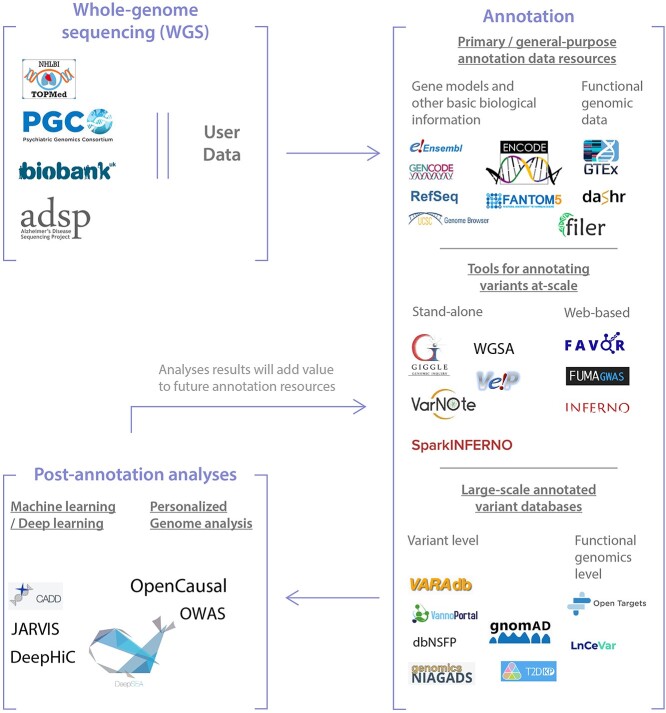

## Introduction

Genetic variants have been shown to associate with various types of diseases and phenotypic traits across populations (1). Many of the genetic variant associations reside in the non-coding, gene-regulatory regions of the genome, such as enhancers and promoters (2,3), and illustrate the importance of their regulatory mechanisms and functional implications. In recent years, whole-genome sequencing (WGS) has emerged as a primary means of capturing and analyzing genetic variants at both the population-scale (4–7) and personal genome level (8–10). WGS can detect millions of variants per genome and capture several variant types, including single-nucleotide variants (SNVs), insertion-deletions (INDELs), copy number variants and structural variants (SVs).

Interpretation and prediction of the functional effects of WGS-identified variants, non-coding variants in particular, remain difficult (11–15). While likely functional candidates among variants in the protein-coding regions can be identified directly by changes to the protein sequences or splicing, interpreting non-coding variants is more difficult as they may affect both genetic and epigenetic mechanisms that impact the gene expression and regulation (e.g. enhancer elements, transcription factor-binding, deoxyribonucleic acid (DNA) methylation and chromatin accessibility) often in a tissue-/cell-type-specific manner (16–18). For rare, low-frequency variants, conventional single-variant genome-wide association (GWAS) testing has limited statistical power—a large number of samples is needed to recruit sufficient carriers. Resolution of GWAS for causal variant finding is further affected by the linkage disequilibrium, where the neighboring variants often display similar associations with the tested GWAS condition. Thus, characterizing effects of non-coding regulatory variants requires the integration of WGS or GWAS results with tissue- and cell-type-specific regulatory activity data and LD information. The goal of this integrated approach is to identify the causal variants, regulatory elements, target genes and specific tissue/cell types they affect.

Population-level GWAS analyses can help to identify trait- or disease-associated variants and loci (19). Follow-up post-GWAS analyses, such as statistical fine-mapping (12), can more precisely identify the causal variants from GWAS-identified loci, while colocalization analyses (20) can identify target genes, and SNV-enrichment analyses can prioritize trait- or disease-relevant tissues and cell types (11). Many of such variant analyses utilize the functional annotation data to provide biological context, identify affected genes and molecular mechanisms and enhance statistical power.

Variant annotations are useful for identifying the relevant variants and prioritizing them for further investigation (21–25). However, the scale and heterogeneity of functional genomics (FG) datasets and genomic annotations necessitate systematic, integrative methods for such functional characterization of WGS genetic variants (11,13,14,26). For example, large-scale projects, such as Encyclopedia of DNA Elements (ENCODE) (27), Roadmap Epigenomics (28), Genotype-Tissue Expression (GTEx) (29) and FANTOM5 (18), have together compiled hundreds of thousands of experimental datasets across >1000 tissues, cell types and biological conditions, each with millions to billions of records across the genome. Additionally, modern population-level WGS studies such as UK Biobank (6) (500 000 individuals with >2500 phenotypes), Trans-Omics for Precision Medicine (TOPMed) (4) (~200 000 individuals; Freeze 9) and specific disease-focused studies, such as Alzheimer’s Disease Sequencing Project (30) and International Cancer Genome Consortium-Accelerating Research in Genomic Oncology (31), all provide extensive WGS data to be probed for the causal or regulatory roles of variants.

**Figure 1:**
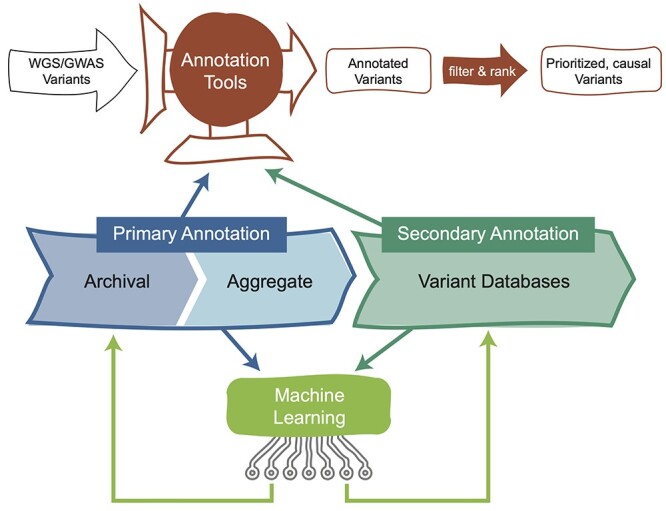
Functional analysis of (non-coding) WGS variants at scale. WGS-identified variants are annotated using a combination of primary and secondary annotation databases, annotation tools and machine learning approaches (see Table 1 and the corresponding Results—Discussion sections for the summary and descriptions of these approaches). Post-annotation analyses are conducted to identify likely candidate, causal variant and to prioritize variants (illustrated in the diagram by filter and rank steps applied to annotated variants). Analysis results can further add to the annotation resources (illustrated in the figure by feedback loops from machine learning-based analyses). **WGS-identified variants:** Variants identified from analysis of user WGS or GWAS data or data retrieved from biobanks, genotyping and sequencing archives and disease-specific sequencing initiatives. **Primary annotation:**  *Archival:* experimentally derived data that are archival in nature, such as nucleotide and protein sequences, regulatory elements identified via WGS that are submitted directly by researchers; *Aggregate:* aggregate databases of harmonized and standardized primary data that are often indexed and associated with an API to facilitate scalable programmatic access (see Common annotation data resources section). **Secondary annotation:** databases of variant annotations curated from the literature or derived from harmonization and analysis of primary data and annotations, often themed with annotations selected to serve specific community needs (see Annotated variant databases section). **Annotation tools:** standalone software or web-based interfaces designed to efficiently query and map genomic features from primary annotation resources and variant annotations from secondary resources against user-supplied lists of WGS-identified variants (see Annotation tools section). **Machine learning approaches:** primarily tools designed for learning from existing data such as annotations, sequence and other features and creating predictive models for characterizing variant effects and functions (see Machine learning approaches section).

To process such large-scale data, scalable methods and computational frameworks have been developed to annotate WGS-identified genetic variants and genomic regions (Fig. 1), including robust and easy-to-use software annotation tools (Annotation tools section), annotation databases (Annotated variant databases section) and experimental data repositories (Common annotation data resources section). Together, these databases and toolkits facilitate the systematic interpretation of hundreds of millions of genotypes across millions of subjects. In this review, to address the challenges of analyzing non-coding variants, we discuss the functional annotation and analysis frameworks for WGS variants in each of these contexts as well as address machine learning prediction-based approaches that leverage these resources to provide insights into variant pathogenicity (Machine learning approaches section). We also discuss future research directions that are needed to overcome the existing challenges and improve the scalability and effectiveness of functional analyses of WGS-identified variants which in turn will serve to improve our understanding of the disease etiology.

## Results

### Common annotation data resources

Interpreting the functional relevance for millions of WGS-identified variants involves interrogating multiple, diverse tissue- or cell-type-specific functional genomic and annotation datasets. If this is the case, individual results across such heterogeneous data sources and data types must be subsequently linked and summarized. These steps are made difficult by the heterogeneity and breadth of data types and experimental assays used to generate these data and the specific tissue and cell-type contexts of individual datasets (26).

Recent, large-scale efforts are directed toward experimentally capturing a variety of `omics data (including transcriptomics, epigenetics, interaction data, proteomics and metabolomics) within single cell- or tissue-specific contexts. Several initiatives have made significant inroads into systematically assembling these data, many of which have been established for more than a decade and have become cornerstones for human genetics research (18,27–29). The ENCODE consortium (27) has generated an extensive collection of primary datasets identifying functional elements (e.g. transcription factor-binding sites, open chromatin regions) by employing a variety of experimental assays, such as chromatin immunoprecipitation with massively parallel DNA sequencing (34), assay for transposase-accessible chromatin using sequencing (ATAC-seq) (35) and DNase-seq (36), across a common set of cell and tissue types. The GTEx project (29) focuses on the cell- and tissue-specific profiling of gene expression and the identification of expression quantitative trait loci (QTL) to link genetic variants with their target genes in the same biological contexts (29). Other examples of these primary annotation resources are provided in Table 1.

Despite the massive effort involved in assembling these large-scale FG datasets, they are still limited in that data are sparse or non-existent for a broad swathe of biological conditions, tissues and cell-types. Recent works, such as EpiMap (37), attempt to remedy this by training models on the available data and by imputing missing data from the available incomplete data. Moreover, due to the sheer size and complexity of these data sources, use of these resources is not straightforward. Methods that use these data need to robustly handle different protocols, different tissues and cell-type contexts at a large scale (see Annotation tools section for a review of such approaches). Additionally, methods are needed to integrate this genomic knowledge with the genetic data in the context of genetic findings and their interpretation (11) (see Annotation tools section).

These needs have led to the development of aggregate databases that curate, integrate and summarize functional data from the primary sources into ready-to-use catalogs of genomic elements important to function or regulation, including RefSeq Functional Elements (38), Ensemble Regulatory Build (39), ENCODE Screen (40), Functional Genomics Repository (FILER) (26) and the FAVOR Essential Database (http://favor.genohub.org/). For example, ENCODE Screen (40) provides a catalog of candidate regulatory elements based on integrating open chromatin, histone marks, transcription factor-binding and related information. The FILER database (26) provides a large-scale collection of harmonized, indexed FG and annotation datasets that can be searched interactively via the web or programmatically queried using an application programming interface (API). The Ensembl regulatory build (39) focuses on annotating transcription factor-binding sites, open chromatin regions, promoters and enhancers.

Overall, current aggregate databases share several limitations and can be further improved in the future, e.g. by capturing tissue sample information at single-cell or individual cell-type resolution and by expanding the coverage for tissues, environmental conditions and developmental stages.

**Table 1:** Primary data resources, annotation tools and databases for WGS variant annotation and prioritization

Name	Description	Objective	Annotates	Access			
				D	S	A	W
**Primary annotation resources**							
ENCODE (27) (https://www.encodeproject.org/)	Public repository for the ENCODE research consortium aimed at identifying all functional elements in human and model organism genomes	Access primary experimental data for tissue- or cell-specific FG	Genomic regions	●		●	
FANTOM5 (18) (https://fantom.gsc.riken.jp/5/)	Public repository for the FANTOM research consortium aimed at identifying regulatory elements in human and model organism genomes	Access primary experimental data for tissue- or cell-specific transcriptional regulatory elements	Genomic regions	●			●
Roadmap (28) (http://www.roadmapepigenomics.org)	Public repository for the Roadmap Epigenomics project for providing access to human epigenomic data	Access primary experimental data for tissue- or cell-specific epigenomics	Genomic regions	●			
FILER^a^ (26) (https://lisanwanglab.org/FILER)	Indexed, searchable database of aggregated and harmonized primary experimental data and gene and variant annotations	Batch query tissue-or cell-specific molecular and FG or sequence feature annotations	Genomic regions	●	●	●	●
EpiMap (37) (http://compbio.mit.edu/epimap)	Database of aggregated and harmonized primary data from ENCODE, Roadmap, and imputed epigenomic datasets	Browse harmonized primary and imputed data and predicted gene or disease regulation for tissue- or cell-specific epigenomics	Genomic regions	●			●
**Annotation tools**							
FUMA (14) (https://fuma.ctglab.nl/)	Interactive web-based functional annotation of GWAS results	Prioritize lead SNVs and annotate function in context of gene models	SNVs,^b^ genes	●			●
SparkINFERNO^a^ (13) (https://bitbucket.org/wanglab-upenn/SparkINFERNO) web-based interface (15,47) (http://inferno.lisanwanglab.org)	Standalone, scalable functional annotation of GWAS results	Annotate and prioritize lead variants in tissue- or cell-specific biological context	SNVs, INDELs, SVs^b^		●		●
Giggle^a^ (44) (https://github.com/ryanlayer/giggle)	Standalone, scalable genomics search engine	Efficiently query and rank genomic loci across user-assembled primary experimental data	Genomic regions		●		
VarNote^a^ (45) (http://www.mulinlab.org/varnote/index.html)	Standalone, scalable functional annotation of GWAS or WGS results in tissue- or cell-specific context	Annotate and prioritize lead SNVs	SNVs		●		●
WGSA^a^ (48), (https://sites.google.com/site/jpopgen/wgsa)	Standalone, scalable annotation pipeline for functional annotation of WGS results	Annotate variants and place in biological context of gene models or regulatory elements	SNVs, INDELs	●	●		
VCFANNO^a^ (49) (https://github.com/brentp/vcfanno)	Standalone, variant annotation pipeline	Annotate variants based on user-assembled primary experimental data	SNVs, INDELs, SVs		●		
VEP^a^ (23) (https://ensembl.org/vep)	Standalone tool for basic variant annotation; plugins for inclusion of third-party annotations	Predict potential variant effect, place in gene or regulatory context	SNVs, INDELs, SVs		●		●
SnpEff^a^ (22) (http://pcingola.github.io/SnpEff)	Standalone tool for basic variant annotation and filtering, sorting, and ranking large result sets	Predict potential variant effect, place in gene context; rank and filter based on annotations	SNVs, INDELs, SVs		●		
AnnoVar (25) (https://annovar.openbioinformatics.org)	Standalone tool for gene- or region-based variant annotation and filtering	Identify variants in gene or genomic regions or identify and filter variants based on annotations	SNVs, INDELs, SVs		●		
RegulationSpotter (50) (https://www.regulationspotter.org/)	Web-based annotation tool	Annotate non-coding variants in a human-readable format for use and interpretation by clinicians	Single or batch SNVs, INDELs				●
**Variant databases**							
Open Targets Genetics (51) (https://genetics.opentargets.org/)	Interactive variant knowledge base and annotation tool linking curated genetic associations and FG data	Prioritize candidate causal variants at trait-associate loci to facilitate identification of potential drug targets	Single gene or SNV	●		●	●
VannoPortal (46)	Comprehensive interactive variant annotation database	Comprehensive context-specific variant annotation (>33 tissues/cell types)	Single SNV or INDEL				●
VARADb (52) (http://www.licpathway.net/VARAdb)	Interactive variant knowledge base linking variants to regulatory elements and potential target genes	Identify or browse regulatory elements proximal to or containing candidate variants	Single or batch SNV	●			●
FAVOR^a^ (http://favor.genohub.org/)	Web-based annotation tool; downloadable database and FAVORannotator R package can be leveraged for WGS annotation	View basic functional annotation of a variant or summarize functional annotation of all variants with a genomic region	Single or batch SNVs and INDELs, by identifier or within a gene or genomic region	●	●		●
Disease-centric knowledgebases^c^	Interactive variant knowledge bases and annotation tools linking disease-specific genetic associations to selected annotations	Identify disease-risk associated variants in gene or genomic regions; browse disease-relevant annotations for candidate variants	Single gene, SNV or INDEL or genomic region, when present, APIs may allow batch lookups			●	●
Curated variant databases (ClinVar) (53,54), (GWAS Catalog) (1), (ADVP) (55)	Interactive variant databases	Identify disease-risk associated variants	SNVs, INDELs, SVs	●			●
Reference variant information (dbSNP) (56), (gnomAD) (57), (TOPMed) (4)	Databases for reference genetic variants and sequencing	Access reference variant information (allele, population frequency, genomic coordinates)	SNVs, INDELs	●		●	●
**Machine learning-based annotation tools**							
Jarvis^a^ (58) (https://github.com/astrazeneca-cgr-publications/jarvis)	Deep learning framework for scoring variant pathogenicity; precalculated genome-wide scores available for download	Rank or filter (non-coding) variants based on predicted pathogenicity scores	SNVs	●	●		
DeepHiC (59) (https://github.com/biocai/DeepHiC)	Deep learning model for predicting variant effects on chromatin interactions	Predict impact of non-coding variants on chromatin interactions and identify potential target genes	SNVs		●		●
CADD, CADD-splice^a^ (60,61) (https://cadd.gs.washington.edu/)	Tools for scoring variant deleteriousness; CADD-splice improves predictions of splicing effects; precalculated genome-wide scores available for download	Lookup or score variant deleteriousness and then use score to rank or filter	SNVs, INDELs	●		●	●
PO-EN (62) (https://github.com/Iuliana-Ionita-Laza/PO.EN/)	R package for presence-only semi-supervised model for predicting regulatory effects of genetic variants	Predict regulatory effects of genetic variants at a GWAS locus	SNVs		●		
MACIE (63) (https://github.com/xihaoli/MACIE)	Unsupervised framework to assess multi-dimensional functional impacts for both coding and non-coding variants	Score variant impact across multiple functional classes	SNVs	●	●		
OpenCausal^a^ (64) (https://github.com/liwenran/OpenCausal)	R package for a sequence-based regression model; packages available for training, scoring and prioritization of variants from GWAS results, leverages WGS	Prioritize non-coding variants by scoring and ranking tissue- or cell-specific impacts in personal genomes	Non-coding SNVs		●		
OWAS (65) (https://github.com/shuangsong0110/OWAS)	R package for predicting chromosome accessibility in personal genomes to prioritize GWAS signals	Prioritize candidate non-coding variants by predicted chromatin accessibility in a cell- or tissue-specific context	Non-coding SNVs		●		
DriverPower (66) (https://github.com/smshuai/DriverPower)	Tool for detecting driver mutations in tumor tissues	Integrate modeled background mutations with functional impact scores to predict coding and non-coding variants in driving cancer	SNVs, INDELs		●		

Access: **D**ownload of data sources or scores (ML) available, **S**tandalone application, **A**PI, **W**eb-based search, analysis or visualizations.

^a^(Red) indicates that resource is scalable or expected to scale to support WGS.

^b^Annotated variant types: SNVs, INDELs and SVs.

^c^Disease-centric knowledgebases [e.g. GenomicsDB (Greenfest-Allen, E., Klamann, C., Gangadharan, P., Kuzma, A., Leung, Y.Y., Valladares, O., Schellenberg, G., Stoeckert, C.J. and Wang, L.-S., manuscript in preparation), https://www.niagads.org/genomics; Type 2 Diabetes Knowledge Portal, https://t2d.hugeamp.org/] (67), (https://www.genomenexus.org/).

### Annotation tools

WGS-identified variants and loci are often evaluated for their biological impact by mapping against known regulatory elements, such as open chromatin, promoter, enhancer or transcription factor-binding sites, at looking for overlap at the variant’s genomic position. As highlighted in the Common annotation data resources section, such assessments involve querying across a large number of massive and often heterogenous functional genomic datasets. Here, we review several popular methods and annotation tools for rapidly searching large data collections to identify the relevant genomic features. A comprehensive listing of these tools is available in Table 1.

#### Genomic feature overlap-based annotation

Several popular tools developed to address these issues and identify overlap with genes and genomic loci of interest or make genome-wide interrogations have been adopted to facilitate the functional annotation of variants using the same primary data sources. For example, Bedtools (41) is a well-established tool that provides a full range of functions for annotating and comparing sets of genomic intervals stored in BED (42), GFF (http://gmod.org/wiki/GFF3) and other standard file formats. Another popular toolkit, Tabix (43), enables the fast retrieval of genomic records in specified genomic loci by indexing position-sorted files.

Whereas Bedtools and Tabix were designed to query genomic features across a limited number of annotation files, more recent approaches, such as Giggle (44) and VarNote (45), attempt to address the scalability problems introduced by WGS and allow efficient querying across larger collections of genomic data. The FILER (26) aggregate FG and annotation database and its API, mentioned in Common annotation data resources section, leverages Giggle to demonstrate the feasibility of designing a large-scale FG and annotation repository with a scalable interface for simultaneously efficiently querying thousands of genomics datasets with billions of genomic features. Similarly, VannoPortal (46), VarNote-based variant portal, provides the ability to dynamically query its collection of functional genomic and annotation data to annotate variants genome-wide.

Differently from such dynamic overlap methods, the Functional Annotation of Variants—Online Resource (FAVOR) (http://favor.genohub.org/), provides a downloadable database of pre-calculated essential annotation scores for a pre-specified set of variants/genomic positions. These annotation scores can be queried directly or using the FAVOR annotator, an R package that facilitates annotation of variants at scale. RegulationSpotter (50), another web-based tool for variant annotation, also provides a region-based regulatory score based on 122 different genomic features and supports single-variant or batch inputs to facilitate the analysis of WGS data. In contrast to these web-based tools, the standalone SparkINFERNO (13) provides a scalable, high-throughput analysis pipeline that retains the flexible, customizable aspects of genomic-overlap tools. Implemented as an extensible, modular analytical system using the Apache Spark (68) distributed computing and data processing framework, it efficiently queries across a broad spectrum of FG datasets. It provides summary text and graphical reports of relevant regulatory elements, tissue contexts and plausible target genes to help prioritize and infer causal variants for lead GWAS signals.

Overall, commonly used annotation tools (e.g. Bedtools, Tabix and Giggle) will benefit from further improvements in computational complexity and indexing/preprocessing strategies to process increasingly larger FG data collections used for WGS variant annotation.

#### Variant aggregation and rare variant analysis

Single-variant analyses have limited statistical power to detect the disease risk-association for low-frequency and rare variants with confidence. To improve the power of association testing for these types of variants, several methods have been developed which aggregate them in biologically relevant regions and then evaluate the association for each of the region. By computing tissue-specific GWAS variant enrichments (11), variant set-based testing can also be used to provide tissue or epigenetic context for the observed associations. However, the output of these aggregation tools depends heavily on how the biologically relevant regions are defined.

STAAR (69) uses fixed sliding windows as well as gene-based windows, prioritized based on annotation principal components, multidimensional summaries of *in silico* variant annotations. This allows STAAR to increase the power for analyzing rare variants in WGS while minimizing type I error rates for both quantitative and dichotomous phenotypes. STAAR is computationally scalable for large WGS, population-scale studies and accounts for the relatedness and population structure using sparse Genetic Relatedness Matrices. On the other hand, eSCAN (70), a recent update to SCANG (71), uses dynamic sliding windows with pre-defined regulatory regions specified as input. Taking another approach, DeepWAS (72) defines variant sets based on their effects on functional units (FUs), which are combinations of cell type, epigenetic feature (transcription factor-binding sites/DNase hypersensitive sites/histone marks) and treatment. The user must input, along with the genotypes, DeepSEA (73) predictions of the effects of variants on FUs, phenotypes and covariates. FunSPU (74) selects variants by integrating multiple association tests and functional annotations to identify genome-wide functionally significant loci. By scaling contributions to the test statistic for specific variant and annotation combinations, FunSPU is adaptive at both the variant and annotation levels. This method increases the statistical power of rare variants, even when data are limited, and addresses the noise introduced by non-informative annotations.

#### Personal genome analysis

While population-level effects of individual genetic variants and their association with the condition of interest are commonly assessed by GWAS analyses, methods for capturing the individual-specific phenotypic effects of genetic variants are only just becoming of interest. Openness weighted association studies (OWAS) (65) is a novel approach which was developed to fill this gap and works by integrating the external LD reference and *in silico*-predicted individual-level chromatin accessibility data to prioritize genes of interest from GWAS analyses of personal genomes. OpenCausal (64) is another new approach that combines personal genomes and tissue-specific transcription factor expression to train an aggregate model to prioritize non-coding variants and predict causal variants. The model is trained on ATAC-seq data from ENCODE samples and then is used to predict variants with the greatest impacts on the chromatin accessibility of regulatory elements based on sequence and transcription factor-binding.

### Annotated variant databases

Recognizing the complexity and computational overhead involved in annotating variants at genome-wide scales, many groups have developed secondary, aggregate databases of derived variant annotations. Most provide some combination of pre-computed annotations (including mapped regulatory elements, predicted causal roles and predicted pathogenicity scores) for genetic variants and basic genomic information (e.g. variant type, closest gene and allele frequencies) (46,52). These databases usually have a web-based front end, allowing researchers, or clinicians and other non-bioinformaticians to easily look up, browse or visually inspect the functional information for a variant or group of variants of interest. Many also provide APIs or standalone software and database downloads which allow researchers to query the resources in batch and integrate data queries into analysis pipelines.

These include resources, such as the web-based front ends for the FAVOR Essential Database (http://favor.genohub.org/) and the VannoPortal (46) previously mentioned in the Annotation tools section, as well as searchable variant reference databases, e.g. dbSNP (56) and gnomAD (57), and general-interest curated variant databases, e.g. ClinVar (53,54) and NHGRI-EBI GWAS Catalog (1)). Also available are more topical resources directed to specific research communities. These include disease-specific annotation resources that annotate at scale sets of variants identified from GWAS or relevant literature sources such as the Type 2 Diabetes Knowledge Portal (https://t2d.hugeamp.org/) or GenomeNexus (67) for cancer-related variants. VariCarta (75) and ADVP (55) provide curated catalogs of variants found in the autism spectrum disorder and Alzheimer’s disease studies, respectively. Open Target Genetics (51) focuses on providing statistical evidence (e.g. QTL, GWAS association and colocalization information) for links between genetic variants and potential drug targets. LincSNP 3.0 (76) documents disease- or phenotype-associated variants in human long non-coding ribonucleic acids (RNAs) and circular RNAs or their regulatory elements and provides online tools for data retrieval and analysis as well as interactive browsing. Additional variant databases and associated access (web, API and downloads) are listed in Table 1.

### Machine learning approaches

WGS-based machine learning approaches constitute another set of important tools available for characterizing the variant effects and functions. Several of the tools and databases mentioned in previous sections depend on some machine learning component, e.g. OpenCausal (64), DeepWAS (72) and EpiMap (37), to train models that impute missing data or predict potential regulatory impacts and prioritize WGS variants. Machine (and by extension deep) learning approaches to variant annotation or functional prediction are usually either semi-supervised or unsupervised. Semi-supervised deep learning models using pseudo-labeling have been shown to have some advantages when working with limited datasets. For example, Jia and others (77) introduce an effective approach that leverages a semi-supervised neural network, using both labeled and unlabeled data, to efficiently identify non-coding mutations in human diseases. In contrast, the recently proposed multi-dimensional annotation-class integrative estimation (MACIE) (63) uses a novel unsupervised framework for synthesizing multiple annotations to predict the likelihood of each variant’s functional impact. Precomputed scores against all variants in the human genome are available for download.

Other standalone tools focus on the issue of cell and tissue specificities of variant regulation. These include GenoNet (78) and PO-EN (62), which both use semi-supervised learning to leverage functionally validated data and chromatin features to predict the tissue-specific function of novel variants. Similarly, TURF (79) uses a random forest model that uses features from functional genomic annotations to compute the tissue-specific regulatory impact scores for sets of variants. Scores for all SNVs from the NHGRI-EBI GWAS Catalog (1) are available for download, and TURF is currently being integrated into RegulomeDB v2.0 (79,80).

Another method, DriverPower (66), utilizes a combination of gradient boosting machine and linear models to predict and prioritize coding and non-coding variants affecting cancer progression in tumor tissues. DeepHiC (59) is an example of a method aimed at predicting a specific (chromatin interaction) functional consequence for variants. This deep learning model combines high-throughput chromatin conformation capture (Hi-C) data (81) and interacting DNA sequence information to determine whether a non-coding variant has a functional impact on chromatin interaction. DeepHiC can also identify the potential target gene affected by the variant.

Machine learning methods have also been developed to address the problem of integration, and summarizing annotations to facilitate the interpretation of annotation results. Most of these approaches integrate annotations into an aggregated pathogenicity or functional score (58,60,61). JARVIS (58) uses sequence data, epigenomic annotations and intolerance to variation to predict pathogenicity, as defined in the ClinVar (53,54) database, with single base resolution. In contrast, CADD (60,61) also predicts pathogenicity but is trained on synthetic data to avoid possible bias in ClinVar submissions. CADD scores are available for all possible human SNVs, and GenoNet, PO-EN and Jarvis additionally allow users to re-train the model with their own data.

## Discussion

The large-scale nature of WGS data, compared with GWAS, allows for the detection of common and rare variants as well as SVs associated with disease at an unprecedented scale. However, interpreting WGS results is challenging due both to the sheer size of the data and the diversity and unharmonized nature of annotation and FG data resources. Attempts to address these issues have resulted in a broad spectrum of databases, annotation tools and integrative machine learning approaches for the interpretation and prioritization of WGS variants. Here, we review and summarize the most recent and most widely adopted toolkits, organizing them by the commonalities in design and purpose.

To date, the majority of methods and resources developed for annotating non-coding variants have focused on SNVs. However, SVs (larger SVs, usually >1 kb in length) also greatly impact the functions of the genes encoded in the genome and are responsible for diverse human diseases (82–85). SVs are gaining more attention, thanks to the development of better detection software and technical advances such as long-read sequencing (86). Therefore, several new methods (87–90) have been proposed to effectively annotate and characterize the functional effects and pathogenicity for the identified SVs.

Although there is a significant increase in the number and variety of FG datasets being generated, widespread use of experimental data-based annotation is often limited by the sparsity of available data for particular tissues, cell types and other genomic features of interest. Further developments of predictive biology approaches that address these gaps in experimental data and annotations (such as the imputation-based approach taken by EpiMap) (37) are warranted.

To efficiently use these annotation resources, efforts need to be taken for newly developed approaches to scale well and take on resources to annotate variants in an efficient way. Currently, standardization of variant annotations, and a gold standard of annotation resources, are lacking, e.g. in terms of standard pipelines for generating annotations, annotation data formats and common interfaces for querying and accessing data. Establishing gold standard annotation resources will be extremely beneficial, as any new tools developed can then be compared with the same reference standards. Additionally, benchmarking experiments need to be performed so that practitioners and users will know what to expect of the performance or running times of new tools in their analyses. These tools should be targeted at leveraging both high-performance computing and cloud environments for efficient processing and analyses but should streamline their use and deployment (e.g. by distributing as Docker or Singularity containers and/or providing flexible programmatic access via APIs or easy-to-use web-based interfaces). Importantly, systematic translation of WGS variant findings to gene/drug targets, while remaining challenging (91,92), represents an important direction for future research as genetic support measurably impacts the success of drug targets (93).

With these improvements, we foresee that more approaches will be available to elucidate the impact of different kinds of non-coding variants (such as singletons and ultra-low frequency variants) on disease etiology and mechanisms.

*Conflict of Interest statement*. None declared.
